# Search for OIE-listed ruminant mycoplasma diseases in Afghanistan

**DOI:** 10.1186/s12917-017-1067-7

**Published:** 2017-05-30

**Authors:** W. Bahir, O. Omar, R.S. Rosales, M. Hlusek, G. Ziay, W. Schauwers, A.M. Whatmore, R.A.J. Nicholas

**Affiliations:** 1 Central Veterinary and Diagnostic Research Laboratories, Kabul, Afghanistan; 2 Animal and Plant Health Agency, Woodham Lane, Addlestone, Surrey KT15 3NB UK; 3 Landell Mills, Trowbridge, Wilts BA14 8HE UK; 4 Consultant, The Oaks, Nutshell Lane, Farnham, Surrey GU9 0HG UK

## Abstract

**Background:**

Little is known about the occurrence of important diseases of ruminants in Afghanistan because of the conflict affecting the country over the last 40 years. To address this discrepancy, ruminant herds in Afghanistan were screened for OIE-listed mycoplasma diseases, contagious bovine (CBPP) and caprine pleuropneumonias (CCPP).

**Results:**

Of the 825 samples from 24 provinces tested for serological evidence of CBPP caused by *Mycoplasma mycoides* subsp.*mycoides*, 20 (3.4%) had ELISA values greater than the positive threshold of 50% though all were less than 55%. Repeat testing of these suspect sera gave values below 50. A smaller number of sera (330) from cattle in nine provinces were also tested by the rapid latex agglutination test (LAT) for CBPP, 10 of which were considered suspect. However, no positive bands were seen when immunoblotting was carried out on all sera that gave suspect results. Serological evidence of *Mycoplasma bovis* was detected in half of 28 herds in eight provinces. The cause of CCPP, *M. capricolum* subsp*. capripneumoniae* was not detected in any of the 107 nasal swabs and lung tissue collected from goats in seven provinces though sample handling and storage were not optimal. However, strong serological evidence was detected in goat herds in several villages near Kabul some of which were over 50% seropositive by LAT and ELISAs for CCPP; immunoblotting confirmed positive results on a selection of these sera.

**Conclusions:**

The data presented here provide a first assessment of the occurrence of the two OIE listed mycoplasma diseases in Afghanistan. From the results of the testing bovine sera from the majority of provinces there is no evidence of the presence of CBPP in Afghanistan. However the samples tested represented only 0.03% of the cattle population so a larger survey is required to confirm these findings. Serological, but not bacterial, evidence was produced during this investigation to show that CCPP is highly likely to be present in parts of Afghanistan.

## Background

Little was known about the current prevalence of animal diseases in Afghanistan until the establishment of the Central Veterinary and Diagnostic Research Laboratories (CVDRL) in 2009 which occupy newly constructed facilities in Kabul, funded by the EU and FAO. Despite considerable challenges within the country, the CVDRL, linked to a network of six regional and twelve provincial laboratories, has established diagnostic facilities for a range of bacterial, viral and protozoal diseases. The formation of an OIE twinning programme with the Mycoplasma Group, Animal and Plant Health Agency (APHA) in the UK provided an opportunity to investigate the presence of important transboundary diseases in particular the OIE-listed contagious bovine (CBPP) and caprine (CCPP) pleuropneumonias caused by *Mycoplasma species*.

Today, CBPP, caused by *Mycoplasma mycoides* subsp. *mycoides*, is believed to be confined to sub-Saharan Africa where it affects at least 26 countries [1]. Little targeted surveillance of the disease has taken place in Asia over the last few decades but freedom from CBPP was declared in India in 1990, China in 1996 and Pakistan in 1997 [1]. There have been no reports of CBPP in Afghanistan or its other neighbours, Iran, Turkmenistan, Tajikistan and Uzbekistan although it is unlikely that these countries routinely monitor for the disease.

The presence of CCPP, caused by *Mycoplasma capricolum* subsp. *capripneumoniae*, has long been suspected in goats in Afghanistan because of reports of outbreaks of disease with similar clinical signs though no laboratory confirmation had been made. Iran reports large numbers of cases annually and there have been detections of CCPP in China, Pakistan and Tajikistan over the last decade [2] so it is highly likely that the disease is present in Afghanistan though the on-going hostilities makes investigation difficult.

The aim of this work was to screen for these two OIE listed diseases in Afghanistan with the support of the OIE reference laboratory CIRAD, Montpellier. It also provided an opportunity to look for other mycoplasmas present in ruminants in particular *Mycoplasma bovis*, an increasingly important cause of bovine mycoplasmosis occasionally mistaken for CBPP [3], and *M. ovipneumoniae,* a frequent isolate from small ruminants affected with atypical pneumonia [4].

## Materials and methods

### Sample collection

Sera and clinical samples were taken randomly from ruminants in most provinces of Afghanistan. Sera for CBPP testing originated from a serum bank established during a national *Brucella* survey carried out in 2012. Samples for CCPP testing were obtained during a joint *Pasteurella*/ *Mannheimia*/ *Mycoplasma* survey in 2013 which covered seven provinces close to Kabul but did not achieve all its objectives because of problems with sample collection and storage from some provinces. In most cases sera were transported successfully and without delay to the CVDRL in cool boxes at approximately 4 °C and stored at -80 °C until all sera was collected and then tested together. All swab and tissue samples were transported as for the sera but tested immediately on arrival. It should also be noted that due to logistical and other problems clinical reports of disease were not comprehensive or considered reliable so were not included in this study.

#### Cattle samples

**Fig. 1:**
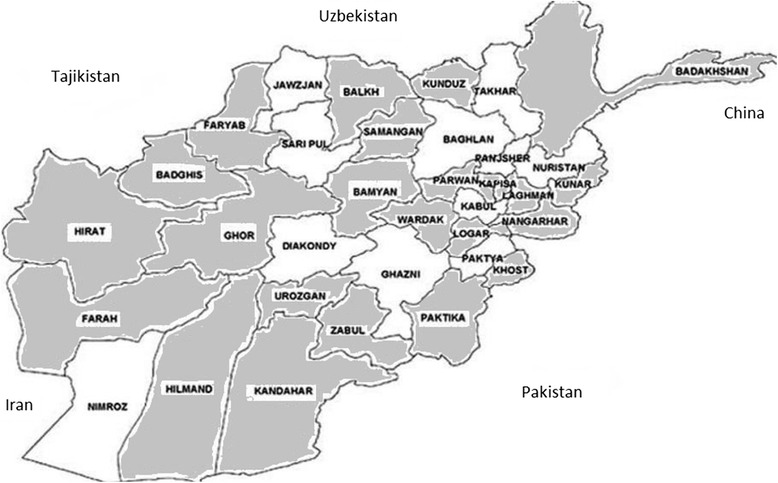
Map of Afghanistan showing (shaded) provinces sampled for CBPP

In total, 825 samples from 79 villages comprising 142 herds were tested. The sera had been collected from 24 of the 34 provinces of Afghanistan (Fig 1); sample sizes varied from 1 to 32 animals per village averaging 7. About 90% of cattle sampled were female and all were over 2 years old.

#### Goat samples

A total of 325 serum samples were obtained from herds in seven provinces in 2013; 88 nasal swabs and 19 lung tissues were taken from adult goats in an additional four provinces where clinical signs suggested the presence of CCPP. Sample size averaged 15 goats per village which sometimes consisted of more than one herd but animals mostly shared the same grazing area.

#### Laboratory tests for CBPP and bovine mycoplasmosis:

The following tests were performed: commercial competitive (c) ELISA (Idexx, France) and latex agglutination test (Bovi-LAT, APHA, UK) for CBPP and indirect ELISA for *M. bovis* [5].

The cELISA was performed as stated by the manufacturer with a cut-off established at 50%; results below this cut-off are considered seronegative for CBPP. Training of staff from CVDRL was conducted by APHA, Weybridge during the OIE twinning programme. The World Reference Centre for CBPP, CIRAD, France conducted quality assurance testing of the CVDRL staff performing the cELISA and also kindly provided the kits; 32 sera of unknown status were sent to CDVRL for QC testing. Results matched exactly those of CIRAD.

Training and reagents for the indirect ELISA for *M. bovis* were provided by the APHA, UK. The test is run routinely at APHA where a cut-off point of 0.3 had been established values greater than this are considered positive.

The latex agglutination test (Bovi-LAT) was obtained from APHA and was performed according to their instructions. Results are scored subjectively after 2 min as: no agglutination: negative; slight agglutination: + suspect positive; agglutination within 2 min: ++ positive; agglutination with 1 min +++: strong positive.

Western blotting was carried out using the type strain PG1 as antigen. Strips were prepared by APHA, Weybridge [6] and dispatched to CDVRL. Sera were diluted 1/50 and applied to the antigen coated strips. For the western blot to be valid, the positive control should develop a distinctive pattern of bands [7]. Samples are compared to the positive control. A positive result is determined by four or more bands of equal size to the positive control. A sample is negative if the result does not follow the distinctive pattern or is not of equal size to the positive control.

#### Laboratory tests for CCPP

*Mycoplasma* culture was carried out on nasal swabs and lung tissue from selected goats in seven provinces [8]. Specific *Mycoplasma* medium was obtained from Mycoplasma Experience Ltd. (Reigate, UK) which was capable of supporting the growth a range of ruminant mycoplasmas including the fastidious *M. c. capripneumoniae*. The following PCRs were used to identify mycoplasmas following 24 h growth in mycoplasma medium: first, a generic mycoplasma PCR was carried out with primers described by Tang et al. [9] using *M. c. capripneumoniae, M. ovipneumoniae* and *M. arginini* as positive controls. If the samples matched the bands of either the *M. ovipneumoniae* or *M. arginini* controls then identification was achieved for these mycoplasmas; however as this PCR does not differentiate the *M mycoides* cluster (which includes *M c. capripneumoniae)* then a *M. mycoides* cluster PCR [10] and specific PCR for *M. c. capripneumoniae* [11] were carried out.

The latex agglutination test (Capri-LAT) was obtained from APHA and was performed according to their instructions. Results were interpreted as for CBPP.

The competitive ELISA (Idexx) was carried out according to the manufacturer’s instructions. In addition, an indirect test for antibodies to CCPP was carried out with concentrated culture-grown antigen prepared at APHA [12]. and sent to CDVRL where a chequerboard titration was carried out to establish optimum concentrations of antigen and peroxidase-labelled IgG conjugate. Antigen and conjugate was diluted 1/200 and 1/2000 respectively. A cut-off point of the mean optical density plus 4 standard deviations was established as 0.3 after testing 100 goat sera from the UK, where CCPP has never been recorded.

The immunoblotting method was adapted from that previously described using the F38 strain of *M. c. capripneumoniae* [6]. Field sera were diluted 1/50 and applied to the antigen-coated strips which had been prepared at APHA and sent to CDVRL for testing. Results were interpreted as for CBPP.

## Results

### CBPP

Of the 825 samples tested by cELISA, 20 (3.4%) had values greater than 50% though all were less than 55% (Table 1). Repeat testing of these suspect sera gave values of below 50. Immunoblotting was carried out on all sera that gave suspect results in the cELISA. No bands of the correct size for CBPP were seen; indeed bands on the strips were either absent, faint or indistinct. However because of the shortages of reagents, none of these suspect sera were tested by LAT or *M bovis* ELISA.

**Table 1:** Serological survey for CBPP and bovine mycoplasmosis in cattle herds in 24 provinces of Afghanistan

Province	No. of herds	No. of samples	No of positives cELISA	No of positives Bovi-LAT	No of positives *M bovis* ELISA
Bamyan	5	35	0	(1)^b^	0 (0)^c^
Kunar	4	32	7^a^	-	-
Kabisa	2	16	3^a^	-	-
Parwan	4	36	0	0	13 (4)^c^
Badakhshan	1	7	0	-	-
Balkh	3	30	0	0	0
Laghman	3	28	0	(1)^b^	18 (3)^c^
Ghor	3	35	0	(6)^b^	30 (3)^c^
Khandahar	2	39	0	(1)^b^	20 (2)^c^
Kanduz	2	18	0	(1)^b^	0
Nangharhar	1	31	0	0	-
Zabul	6	78	2^a^	0	20 (2)^c^﻿
Patkya	6	64	2^a^	-	-
Khost	6	113	1^a^	-	-
Helmand	3	84	0	-	-
Wardak	6	29	1^a^	-	-
Logar	4	33	1^a^	-	-
Daykondi	5	26	1^a^	-	-
Farah	4	29	1^a^	-	-
Herat	2	19	1^a^	-	-
Badgis	4	33	0	-	-
Samangan	1	7	0	-	-
Uruzgan	1	3	0	-	-
Fariab	1	0	0	-	-
Total	79	825	20	0	101 (14)

^a^Negative on repeat testing and by western blot

^b^Sera giving suspect results on LAT but negative by western blot

^c^Herds seropositive for *M bovis*

- not tested

LAT was carried out on 330 sera from 29 herds in 9 provinces, 10 sera had scores of 1+, which is considered suspect (Table 1). However these sera were tested by immunoblotting and found to be negative.

The indirect ELISA for *M bovis* was carried out on 272 sera from 28 herds in eight provinces. In all, 14 herds contained seropositive cattle, seven of which were over 50% seropositive (Table 1). No seropositive cattle were seen in the provinces of Bamiyan, Kanduz and Balkh though numbers of cattle tested were relatively few. No clinical reports were received from the herds tested.

### CCPP

*Mycoplasma c.capripneumoniae* was not detected in any of the 107 nasal swabs and lung tissue cultured and tested by PCR for mycoplasmas. *M. ovipneumoniae* and *M. arginini* were detected in 55 and 9 samples respectively (data not shown). A total of 16 of 19 nasal swabs were positive for *M. ovipneumoniae* in one village in Kunar Province but no clinical reports were available on goat health.

Of the 21 villages sampled in seven provinces, seven were seropositive by cELISA, 8 were seropositive by LAT and 12 by iELISA (Table 2). Five of the villages were positive by all tests; two of these, Starbik and Arbabdara, were from Kabul province and were 47 and 40% by cELISA, 47% and 83% seropositive by LAT and 73% and 63% seropositive respectively by iELISA. A selection of 10 sera from different herds, positive by indirect ELISA, were tested by immunoblot. Of these six gave strong bands of the same size as the control serum.

**Table 2:** Serological testing for CCPP of goat herds in 8 provinces in Afghanistan

Province	Villages	No of samples	No of positives Capri-LAT	No of positives iELISA	No of positives cELISA
Bamyan	Akhzarat	10	3	0	2
Kunar	Shahedan	9	0	0	2
Kabul	Mohammady	3	1	1	0
	Dasht said	7	0	0	0
	Starbik	30	14	22	14
	Arbabdara	30	25	19	12
	Khoshab	31	3	4	1
	Qasaban	25	0	0	0
Kabisa	Qasaba	18	0	0	0
	Dara Dasht	6	0	1	0
Parwan	Kampany	4	0	0	0
	Parwan	4	0	0	0
	Sabat	27	0	0	0
	Polemoghulan	30	0	0	0
Badakhshan	Said Khil	16	0	2	0
Balkh	Sanjed Dara	19	0	3	0
	Barek Ab	13	0	4	0
	Qala-e-Dana	11	0	3	0
	Dahandara	12	4	1	7
	Gozar Marpol	8	2	2	1
	Arghawan	12	1	2	0^a^
Total 7	21	325	53 (8)^b^	64 (12)^b^	38 (7)^b^

^a^Only 2 samples tested by cELISA

^b^number of positive villages

## Discussion

The data presented here provide a first assessment of the occurrence of the two OIE listed mycoplasma diseases in Afghanistan. From the results of the testing of cattle sera from the majority of provinces there is little evidence of the presence of CBPP in Afghanistan. The sample tested represented only 0.03% of the cattle population but has a good statistical chance of detecting disease at a prevalence of at least 1%. One province, Kunar, contained a high number of suspect ELISA-positive samples (nearly 20%) spread amongst four herds on first testing but these were found to be negative on retesting and by immunoblotting. Publicity including talks and posters have been generated by the CVDRL staff during the twinning process with APHA describing the typical clinical signs and lesions of CBPP and will help the local veterinary staff to recognise the disease should it be present.

However from a smaller survey of eight provinces, it is clear that *M. bovis* is present with most herds examined containing seropositive animals, some up to 90% positive. Only the five herds tested in Bamiyan Province were negative. This finding is not surprising given that only one cattle-rearing country to date, New Zealand, remains *M bovis*-free. Unfortunately, the presence of *M bovis* could confound attempts to declare Afghanistan free of CBPP as gross lesions can sometimes be very similar [13]. Furthermore, serological cross-reactions have been reported between the two mycoplasmas during the CBPP eradication campaign in Portugal [3] although there was little evidence of it in the present study as only two false positives were seen during the testing of 14 herds which were seropositive to *M bovis*. Interestingly the false positive rate of the cELISA for CBPP was just over 3% using the strict cut-off of 50% on first testing but 0% if 55% is used. All sera gave values below 50% on repeat testing. The use of the highly specific immunoblotting test provided confirmation of the negative status of all suspect sera.

The presence of CCPP in Afghanistan has long been suspected due to its presence in neighbouring countries and from clinical reports from the field. Indeed the main reason for the twinning programme was to provide reagents, equipment and training to enable detection of this most fastidious of mycoplasmas. To this effect serological, but not bacterial, evidence was produced during this investigation to show that CCPP is highly likely to be present in parts of Afghanistan.

Of the goat herds in seven provinces, strong serological evidence was seen in Kabul and Balkh where many sera were positive by ELISAs and LAT; in a smaller sample both the cELISA and LAT were positive in several goats in Bamyan province. Some differences were seen in the sensitivities of the tests with cELISA detecting fewer positive sera than the other tests but this is to be expected as the cELISA is generally considered more specific [14]. Unfortunately the causative mycoplasma *M. c.capripneumoniae* could not be detected or isolated from nasal or lung samples probably due to poor sample handling, storage and time taken for the samples to reach the CVDRL in Kabul. Predictably, the less fastidious mycoplasmas such as *M. ovipneumoniae*, an occasional cause of respiratory disease in small ruminants, and the opportunist *M. arginini* were detected, mostly in nasal swabs from goats in most provinces especially in a herd in Kunar where there had been no serological evidence of CCPP.

## Abbreviations

(c) ELISA: (competitive) enzyme-linked immunosorbent assay
CBPP: contagious bovine pleuropneumonia
CCPP: contagious caprine pleuropneumonia
LAT: latex agglutination test
PCR: polymerase chain reaction
